# Exploring Endovascular Photo-Activated Ablation (EPA) for Downstaging Locally Advanced Pancreatic Cancer: A Proof-of-Concept Study in the Normal Porcine Model

**DOI:** 10.3390/cancers17203340

**Published:** 2025-10-16

**Authors:** Alain García Vázquez, Juan Manuel Verde, Fanélie Wanert, Irene Alexandra Spiridon, Axel Schmid, Tina Saeidi, Lee L. Swanstrom, Stephen G. Bown, Lothar Lilge, Arjen Bogaards

**Affiliations:** 1 IHU Strasbourg, Institute for Image-Guided Surgery, 67091 Strasbourg, France; 2 Department of Pathology, “Grigore T. Popa” University of Medicine and Pharmacy, 700115 Iasi, Romania; 3 Institute of Radiology, University Hospital Erlangen, 91054 Erlangen, Germany; axel.schmid@uk-erlangen.de; 4 Department of Medical Biophysics, University of Toronto, Toronto, ON M5G1L7, Canada; 5 Research Department of Targeted Intervention, Division of Surgery and Interventional Science, University College London, London W1W 7TY, UK; s.bown@ucl.ac.uk; 6 Princess Margaret Cancer Centre, University Health Network, Toronto, ON M5G1L7, Canada; 7 Faculty of Health Science, Laser Research Centre, University of Johannesburg, Doornfontein, Johannesburg P.O. Box 17011, South Africa; 8 Vascular Oncology Biotechnologies B.V., 6525 EC Nijmegen, The Netherlands; info@vascularoncology.com

**Keywords:** endovascular photo-activated ablation, downstaging vascular involvement of locally advanced pancreatic cancer, verteporfin

## Abstract

Background: Pancreatic cancers can involve large blood vessels early, making complete resection technically challenging or impossible. A minimally invasive treatment that clears vessels from encasing tumours could potentially enable curative surgery. We hypothesise that Endovascular Photo-activated Ablation (EPA) of perivascular tumour tissue can create a necrotic zone free of viable tumour between cancer and blood vessels, through which the tumour could be resected. Methods: A dose escalation study was conducted in the normal porcine model (*n* = 7). Under general anaesthesia, the animals were given a photo-activated drug and photo-activation was provided by a prototype balloon catheter, positioned in a major blood vessel within the pancreas, under angiographic guidance. Contrast-enhanced CT scans were undertaken prior to and 1, 2, or 7 days following ablation. The animals were euthanised and the exposed tissue excised en bloc for histological examination. Results: Five animals were euthanised after 2 days. On post-mortem, the histology confirmed necrotic pancreas in the perivascular zone, which increased from zero to 15 mm around the treated vessel, for increasing drug doses. Treated arteries showed necrotising arteritis, without evidence of perforation or obstruction during the observation period, although one animal was euthanised at 1 day, due to technical endovascular device issues and obstruction. The lowest-dose animal euthanised at 7 days showed no lesions on pathology. Conclusions: These proof-of-concept results demonstrate that EPA can produce pancreatic perivascular necrosis in a large animal model. In a pancreatic cancer abutting a major blood vessel, this procedure may be able to create a zone free of viable tumour, potentially rendering these cancers operable, while preserving vessel integrity. These findings support further research activities towards clinical translation.

## Simple Summary

Pancreatic tumors often grow around major blood vessels, making surgery impossible for about one in three patients. A treatment capable of clearing tumor involvement from these vessels would represent a significant clinical advance. In the normal porcine model, we tested a novel approach that administers a light-activated drug and then delivers laser light from within an artery or vein. We have shown that this technique, called Endovascular Photo-activated Ablation (EPA), can safely kill a thin layer of the pancreas next to a major blood vessel, while preserving the integrity and function of the blood vessel. The concept is that in the case of a pancreatic cancer touching the blood vessel, in suitable patients, EPA could create a zone of dead cancer tissue through which a surgeon could safely separate the main mass of viable cancer from the blood vessel to enable complete removal of the cancer without damaging the blood vessel. Further laboratory studies are required to refine the technique and ensure that there are no long term complications. Provided these go well, pilot clinical studies can be planned relatively soon. If proven to be safe and effective, this approach may convert previously inoperable pancreatic cancer patients into surgical candidates with the potential to extend their survival.

## 1. Introduction

Pancreatic cancers are aggressive and tend to involve large blood vessels in the region early, making R0 resection technically difficult or impossible. Further, surgery remains the only potential cure for this disease, which affects over 500,000 patients annually worldwide [1]. Approximately 40% present with locally advanced pancreatic cancer (LAPC), rendering them ineligible for surgery due to vascular involvement [2] or necessitating complex resections with vascular reconstruction. A therapy capable of clearing tumour involvement from vessels could downstage patients and improve the prospects for surgery and longer survival.

Conventionally, chemotherapy and radiotherapy are used for downstaging, with some studies reporting improved survival from 13–16 to 35–40 months [3,4]. However, this comes at the cost of local and systemic toxicities, with the typical treatment lasting weeks or months and benefiting only a minority. Minimally invasive alternatives with shorter durations have been proposed [2,5,6], including radiofrequency, microwave, and cryoablation, irreversible electroporation, electrochemotherapy, brachytherapy, high-intensity focused ultrasound, photothermal, and photodynamic therapy. These are typically delivered interstitially via needle applicators inserted under CT or endoscopic ultrasound guidance. However, accurately ablating tissue adjacent to vessels remains challenging due to anatomical complexity and the need to preserve vascular integrity.

We hypothesise that perivascular ablation may be more effective via the endovascular route, as long as this does not lead to any unacceptable compromise of vessel function.

Endovascular heat-based modalities (laser-thermal, radiofrequency, microwave) risk vessel collapse and occlusion, with effects including intimal and medial disintegration, constriction, carbonisation, and perforation [7,8]. Damage to elastin and collagen—key components of the extracellular matrix—must be avoided [9,10].

Non-thermal approaches may better preserve vessel structure [11,12,13]. Cryotherapy and pulsed electrical field ablation (irreversible electroporation) are used in cardiac applications and can affect the full thickness of vessel walls [14]. However, delivering sufficient energy beyond the vessel wall without heating it remains technically difficult [15,16]. Similarly, cryotherapy via balloon catheters appears limited by technical complexity and prolonged treatment times [17,18,19].

Ionising radiation (e.g., brachytherapy) and photodynamic therapy (PDT) are promising non-thermal options deliverable via the endovascular route. Brachytherapy has delayed effects, with visible response only weeks to months after treatment [12,13,20,21]. PDT induces immediate necrosis, detectable by CT or MRI within 48 h [22,23,24]. We introduce Endovascular Photo-activated Ablation (EPA) as a novel PDT-based approach for tumour ablation via the vessel lumen, aiming to downstage LAPC.

PDT for pancreatic cancer has been studied for decades. It involves systemic administration of a photo-activated drug, followed by activation with low-power red or near-infrared light. The mechanism of action is photochemical, not thermal, generating cytotoxic reactive oxygen species that induce apoptosis and necrosis [25,26,27].

Phase I/II studies in 65 patients with pancreatic or ampullary cancer [24,25,27,28,29,30,31] have shown that needle-based PDT can produce dose-dependent necrosis (0.5–4.1 cm diameter) without pancreatitis or pancreatic fluid leakage. Necrosis in normal pancreas heals safely [23]. Three patients from separate studies were downstaged and underwent surgery [23,31]. In Huggett et al., one of 15 patients underwent a Whipple’s procedure after PDT and survived 30 months, while the others had a median survival of 9.5 months [32]. However, successful needle placement near vessels was largely serendipitous. Delivering light directly via the vessel could improve precision.

Photo-activated ablation using endovascular irradiation has been extensively studied for vascular diseases like restenosis and atherosclerosis. At least five Phase I trials [33,34,35,36,37] and numerous preclinical studies [38,39,40,41,42,43,44,45,46,47] demonstrated feasibility in large vessels without causing stenosis, thrombosis, aneurysm, or rupture, using various photosensitisers including Verteporfin [48,49,50] . However, these studies targeted the vessel wall, not perivascular tumour tissue, with the goal to widen narrowed blood vessels and restore normal blood flow, without causing downstream embolisms.

It remains unknown whether significant perivascular necrosis can be achieved from within the vessel without compromising vascular integrity and function.

Given that most pancreatic cancers are diagnosed late and often abut major vessels, this study investigates whether EPA, using near-infrared light delivered endovascularly, can induce pancreatic necrosis adjacent to vessels without unacceptable effects on the structure or function of the vessel itself. This approach aims to create a zone free of viable tumour, potentially enabling surgical resection

## 2. Methods

### 2.1. Animal Model

We chose to use the normal porcine model, as pigs have comparable anatomy and overall size to humans in the region around the pancreas. The breed used was sus scrofa domesticus, weighing circa 40 kg, sourced from breeding farms in the Strasbourg area. The animals were group-housed and acclimatised in an enriched environment, respecting circadian cycles of light-darkness and with controlled humidity and temperature conditions. The animals were pellet-fed twice a day (piglet diet Lorial/Costal 10–20 g/kg per day) and fasted for 24 h before anaesthesia with ad libitum access to water.

### 2.2. Photo-Activated Drug

Verteporfin was selected as the photo-activated drug due to its reported uptake in normal pancreatic tissues [26] and its use in published clinical pancreatic cancer studies. It is known that necrosis can be produced in normal pancreas and that it clears from the body within one to two days [23,24]. It was administered intravenously over 10 min using a syringe pump at increasing doses of 0.4-3.2 mg kg^−1^, 60–90 min prior to irradiation, as listed in Table 1.

### 2.3. Light Delivery

**Figure 1:**
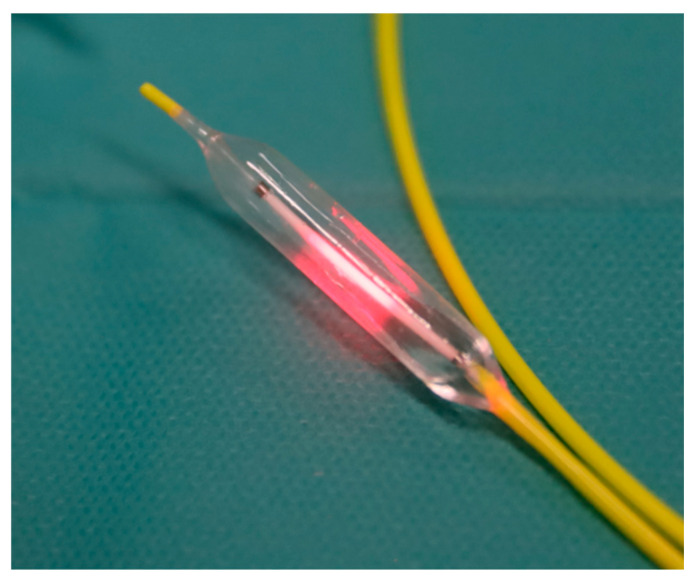
The prototype endovascular irradiator system consisted of a 6 Fr. catheter with a 3 cm long, non-compliant balloon featuring radio markers and a cylindrical optical fibre with a 1cm diffuser tip.

A prototype endovascular near-infrared delivery system was developed as part of the study (Vascular Oncology Biotechnologies B.V., Nijmegen, The Netherlands). It comprised either an 8 Fr. catheter with a 1 cm long compliant balloon or a 6 Fr. catheter with a 3 cm long non-compliant balloon (Figure 1), both having radio markers. The balloons were made of materials with low absorption of near-infrared laser light, and the diameters varied from 4 to 10 mm to accommodate different vessel sizes. Centrally within the balloon is an optical fibre with a 1 cm diffusing tip, coupled to a 1.8 W diode laser, emitting 690 nm light. An integrating sphere, cross-calibrated against a National Institute of Standards and Technology (NIST) traceable power meter, measured the delivered output power before and after irradiation.

Using the Seldinger technique, the transhepatic or transfemoral access route was used for veins and arteries, respectively. The prototype balloon catheter was positioned over a guidewire in either the splenic vein, splenic artery, or superior mesenteric artery under angiographic guidance. Contact of the vessel with the pancreas at the placement site of the ablation catheter was confirmed using preoperative multi-phase contrast-enhanced CT, and the vessel diameter was measured to ensure accurate sizing of the balloon and to prevent overdilation. At 60–90 mins after infusion of the light-activated drug, the ablation balloon catheter was inflated within the target vessel, and the appropriate light dose was delivered.

### 2.4. PDT Dosimetry

Accurate and reproducible tumour clearance from the vasculature using EPA will likely require robust treatment planning, intra-procedural guidance, and post-treatment verification. Our group has initiated the development of software tools capable of simulating anticipated treatment effects, with similarities to radiotherapy treatment planning systems. Previous dose-escalation studies by Huggett et al. and Hanada et al. [23,24] investigated Verteporfin-mediated interstitial photoactivation in patients with pancreatic cancer, employing light doses ranging from 5 to 50 J·cm^−1^ of the diffusing fibre tip and a Verteporfin dose of 0.4 mg·kg^−1^. Based on this data, we performed Monte Carlo simulations to translate needle-based photo-activation parameters to an endovascular light delivery. These simulations informed the initial dose range applied in the present porcine study.

Furthermore, EPA threshold doses were estimated based on observed necrotic tissue responses. As the comprehensive dosimetric analysis is beyond the scope of this manuscript, the findings will be detailed in a forthcoming separate publication [51].

### 2.5. Experimental Procedure

The study was conducted and reported in accordance with the ARRIVE 2.0 guidelines for animal research. Seven normal animals were treated.

All procedures were performed under general anaesthesia in the experimental operating rooms at the IHU Strasbourg. Animals were pre-anaesthetised by i.m. injection of azaperone 1–2 mg∙kg^−1^ (Stresnil^®^ ElancoHuningue, France) combined with zolazepam + tiletamine 5–10 mg∙kg^−1^ (Zoletil^®^ Virbac, Carros, France) prior to being transferred to the operation room. Anaesthesia was induced using i.v. propofol 2–4 mg∙kg^− 1^ (Propomitor^®^, Osalia, Paris, France) and rocuronium 1–2 mg∙kg^−1^ (Esmeron^®^, MSD, Puteaux, France), followed by tracheal intubation. General anaesthesia was maintained with inhaled isoflurane 2% (Isoflo^®^, Zoetis, Châtillon, France) in a mixture of oxygen and air. Controlled ventilation was achieved using the Primus^®^ system (Dräger, Lübeck, Germany), body temperature and vital parameters were monitored throughout the procedure (Maglife Serenity RT1^®^, Schiller, France).

The antihistamine dexchlorpheniramine (Polaramine^®^ 2 mg, Bayer Healthcare, Lille, France) was administered orally twice daily starting the day before EPA to prevent a potential allergic reaction to the photo-activated drug. Additionally, slow bolus i.v. injections of 3 mg∙kg^−1^ of methylprednisolone (SoluMedrol^®^ 120, Pfizer, Paris, France), a corticosteroid, plus 5 mg dexchlorpheniramine (Polaramine^®^ 5 mg∙mL^−1^, Bayer Healthcare, Lille, France) were administered 20 min before i.v. administration of the photo-activated drug.

Intraoperative analgesia was ensured by injection of buprenorphine 0.01 mg∙kg^−1^ i.v. (Bupaq^®^, Virbac, Carros, France). In addition, the animals received an i.m. single dose of nonsteroidal anti-inflammatory, meloxicam 0.4 mg∙kg^−1^ (Emdocam^®^, Axience, Pantin, France) and antibiotics, amoxicillin/colistine 10 mg∙kg^−1^ and 25,000 UI∙kg^−1^ , respectively (Potencil^®^, Virbac, Carros, France).

On the day of treatment, initially, the pancreas was visualised on a baseline CT to plan the procedure. The diameters of the target vessels having contact with the pancreas were measured (Table 1), and a 3D vascular roadmap was obtained using 3D Slicer (www.slicer.org). Under angiography guidance, and using standard interventional techniques, including guidewires and guiding catheters, the prototype near-infrared irradiating balloon catheter was deployed into the target vessel according to the CT-based 3D vascular roadmap.

### 2.6. Follow-Up

During recovery, the animals were closely monitored under veterinarian supervision (F.W.) and kept in a dimly lit room to avoid skin photosensitivity.

After 1-, 2-, or 7-days, animals were sedated by i.m. injection of azaperone 1–2 mg∙kg^−1^ (Stresnil^®^ Elanco, Huningue, France) combined with zolazepam + tiletamine 5–10 mg∙kg^−1^ (Zoletil^®^ Virbac, Carros, France), and contrast-enhanced CT scans were repeated. This was followed by a lethal i.v. injection of pentobarbital 40 mg∙kg^−1^ (Euthoxin^®^, Osalia, Paris, France).

Images were evaluated for evidence of reduced radiological uptake of contrast medium around the treated vessel, indicative of the extent of necrosis (A.S.).

To verify the feasibility of surgery post-ablation, pancreatic surgeons resected the pancreas en bloc after animal sacrifice using the tissue plane along the treated vessel, categorising the differences compared to normal tissues in all animals using a visual analogue scale (VAS): (1). surgery more difficult, (2). surgery moderately more difficult, (3). no difference, (4). surgery moderately easier, (5). surgery easier. Some sections were taken for histology in the plane perpendicular to the axis of the blood vessel to give an overview of all the tissues from the blood vessel to the pancreas.

## 3. Results

### 3.1. Procedure

The procedure was undertaken on 7 normal animals. In the first animal, the splenic artery was irradiated first using the 8 Fr prototype with a compliant occlusion balloon (i.e., one that increases in diameter with increasing pressure). The operator found the device unsuitable to handle in terms of catheter thickness and stiffness. Moreover, the compliant balloon made it impossible for the interventionalist to control the balloon diameter. This led to poor manoeuvrability through vessel curvature, over-manipulation within, and over-distention of the vessel. The day following treatment, this animal looked unwell. A further CT showed that the splenic artery was obstructed, necessitating sacrifice at 1 day post-ablation. Post mortem, at the site of irradiation, a thrombus was found within the treated splenic artery, which otherwise appeared normal and intact upon visual inspection. As there was no evidence of any EPA effect on the arterial wall, this problem was considered most unlikely to be related to the light delivery. No such problems were encountered in any of the subsequent treatments, all of which were performed with the revised 6 Fr light delivery device prototype. It is recognised that oversizing is associated with increased dissection rates and compromised patency [52,53], and together with a relatively stiff catheter and both over-manipulation and overdistension, it was concluded that these factors were the most likely cause of obstruction.

In the same animal, the SMA was treated, 10–15 min after the splenic artery, using the 6 Fr. catheter with a non-compliant balloon (i.e., having a fixed diameter over a wide pressure range). This was found to be easier to handle due to the increased flexibility of the thinner catheter and the interventionalist’s improved control over the balloon diameter. The SMA remained patent, and a necrotic lesion was observed on both CT and pathology 1 day after treatment. Aiming to proceed with caution, the next treated animals received a single ablation via the splenic artery (*n *= 1) or vein (*n* = 5), the latter since the veins often had better contact between the vessel and pancreatic parenchyma on the planning CT. To prevent over-dilation, each received a single ablation with the 6 Fr catheter with a non-compliant balloon, combined with careful matching to the measured target vessel diameter as assessed on CT imaging. There were no significant intra- or post-procedural problems in subsequent animals; they all tolerated the procedure well.

The first animal in the venous group, treated at the lowest drug dose, showed a possible small oedematous lesion on the 2-day CT. It was allowed to survive for 7 days, but no lesion could be identified on pathology. On the 2-day CT scans, the later animals all demonstrated a hypodense lesion in the perivascular region of the pancreatic parenchyma. On histology, these areas were confirmed as necrosis, the extent of which increased with the drug dose used. There was no evidence of perforation or bleeding in any of these treated vessels. Minor problems included the abdomen of one animal being accidentally exposed to surgical lights shortly after photosensitiser injection, resulting in a skin burn during recovery. Care was taken thereafter to avoid exposure to direct operating room lights. There was no clinical evidence of pancreatitis (anorexia, fever, or malaise).

### 3.2. Radiology

On the CT scans, the EPA-induced lesions around treated arteries and veins could be seen as low attenuating, hypo-perfused areas in the perivascular region extending into the pancreatic parenchyma. The results are shown in Table 1.

**Figure 2:**
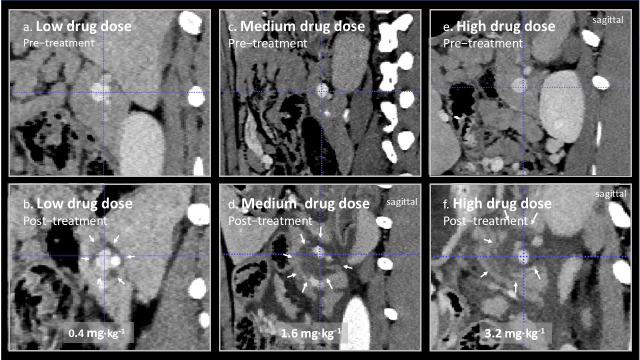
(**a**–**f**) Contrast-enhanced CT scans of treated splenic veins (blue dotted cross-hair) were taken before and 2 days after EPA. Areas of reduced uptake of contrast after treatment are marked by small arrows. The changes in animal 3 treated at a low Verteporfin dose of 0.4 mg∙kg^−1^ (**a**,**b**) are small, possibly perivascular oedema, and of questionable significance, but are of increasing size when the Verteporfin dose was increased to 1.6 mg∙kg^−1^ animal 5 (**c**,**d**) and 3.2 mg∙kg^−1^ (**e**,**f**), in animal 7. Lesions seen on CT were confirmed on histology to be due to pancreatic necrosis. The light dose was nearly constant for these three animals at 377, 311, and 321 J∙cm^−1^, for animals 3, 5, and 7, respectively (Table 1).

In Figure 2, representative images from treated splenic veins are shown in the plane perpendicular to the axis of the treated vein, below an image of the same area prior to treatment for animal 3 (low drug dose), 5 (medium drug dose), and 7 (high drug dose). Perivascular oedema was observed at all doses. In animal 3, it is doubtful weather there was any effect beyond oedema, but circumferential perivascular necrotic margins of increasing size were seen as the drug dose was increased.

### 3.3. Feasibility of Surgery Post-Ablation

During surgical removal of the pancreas at postmortem, the surgeon categorised perivascular dissection in all animals as moderately more difficult, based on the surgeon’s expertise as compared to vessel dissection in the pancreas as part of conventional surgical practice. This was independent of the extent of necrosis.

### 3.4. Histopathological Examination of Pancreatic Specimens

**Figure 3:**
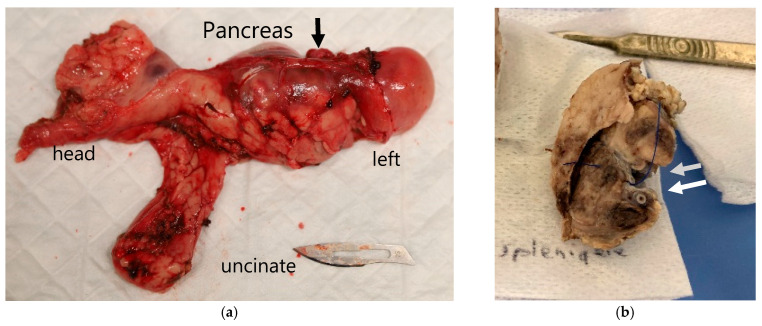
Macroscopic views of the excised pancreas of animal 7 treated at the highest verteporfin dose of 3.2 mg·kg^−1^. (**a**) The entire pancreas was resected en bloc immediately after excision, with a dark area of necrosis in the necrotic area (black arrow). (**b**) demonstrates dissected tissue after formalin fixation, showing the treated splenic vein (top grey arrow) and the artery (bottom white arrow) in cross section, with darkened areas of necrosis in the perivascular pancreatic tissues.

On gross pathological examination of the excised specimens that included the target vessels and the surrounding pancreatic parenchyma, the region of necrosis was identified as a non-structured, reddened, oedematous area within the pancreatic parenchyma, whereas the treated vessels appeared macroscopically normal. Examples are shown in excised tissue before and after formalin fixation in Figure 3a,b.

**Figure 4:**
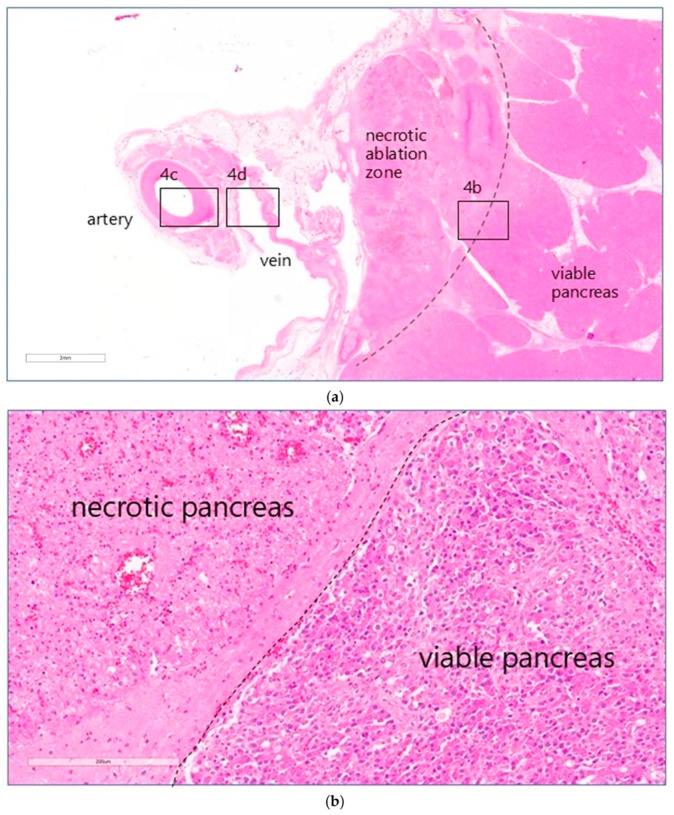

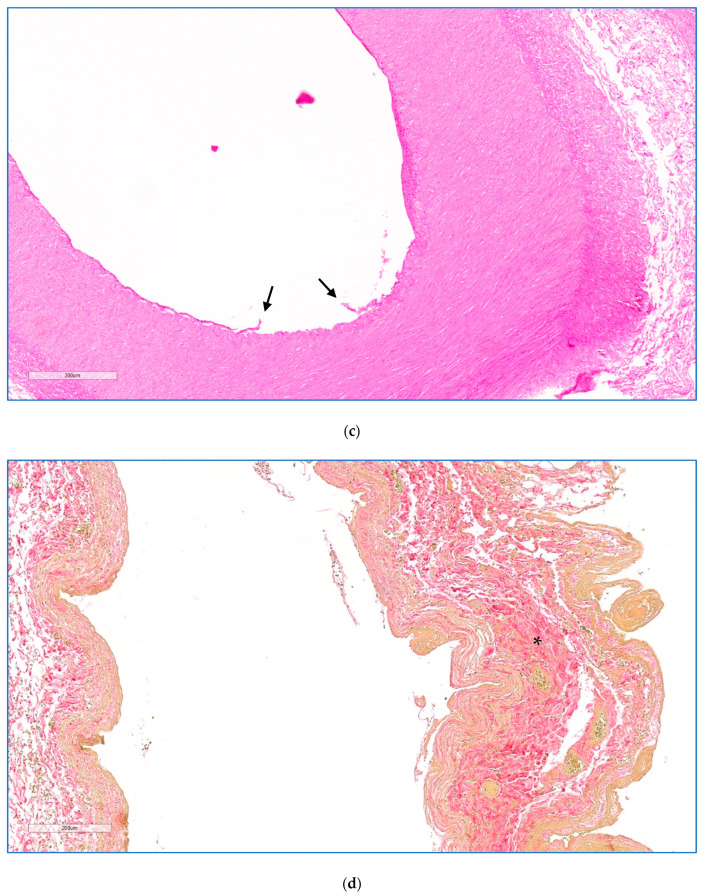
(**a**) Histology of animal 5 treated at a dose of 1.6 mg·kg^−1^. Showing an H&E image with a low-magnification overview (3 mm scale) encompassing the treated splenic vein, splenic artery, a necrotic margin of perivascular necrosis extending into the pancreas, and a sharply demarcated necrotic zone boundary (dotted line), beyond which the pancreatic parenchyma is normal. The perivascular necrotic zone was characterised as having patchy necrosis of pancreatic lobules, with associated cytosteatonecrosis extending into the septa, microthrombosis, haemorrhage, and abundant inflammation rich in neutrophils and macrophages. Higher magnifications of the artery, vein, and pancreas are shown in (**b**–**d**). (**b**). High power image of pancreas showing sharp demarcation (dotted line) between necrotic pancreatic tissues on the left (resulting from EPA) and viable pancreas on the right (H&E, scale 200 μm). (**c**) High power image of the splenic artery (animal 2) showing severe, extensive necrosis of the endothelial lining, endothelial cell detachment (arrow), endothelial cell degenerative change, and marked degeneration of the deep layers. (HE, scale 200 μm). (**d**) High power image of a treated splenic vein, showing collagenisation of the wall (*), with atrophy of the muscular media, and mild transmural inflammatory infiltrate associated with focal ulceration of the endothelium (EVG, scale 200 μm).

On microscopic examination, areas of coagulative necrosis were seen in the pancreas of all animals, except animal 3. These were often patchy and sometimes more extensive, particularly in the animals that had received the higher drug doses. An overview of the entire treated area of animal 5 treated at a dose of 0.8 mg·kg^−1^ is shown in Figure 4a. The distance in mm between the outer edge of the treated vessel and the zones of necrosis in the tissue from each animal was measured (Table 1). At high magnification, the interface between necrotic and morphologically viable tissue was identified as a distinct line (Figure 4b). No definite evidence of necrosis was seen in the pancreas of animal 3, treated at the lowest drug dose.

### 3.5. Histopathological Examination of Blood Vessels

Full-thickness effects were seen in the walls of all vessels examined 2 days after treatment. Microscopic examination of hematoxylin and eosin (H&E) and Elastic Verhoeff Van Gieson (EVG) stained sections was performed (I.A.S.) to evaluate the morphology of the vessel wall (integrity, aspects of the media and endothelium) and the extent of the perivascular pancreatic tissues undergoing necrosis. The results are listed in Table 1. In all animals, except for the one treated at the lowest dose, the endothelium displayed reactive change, erosion/ulceration, or detachment from the basal membrane. The vessel wall showed necrotising arteritis, leading to a thickened intima with atrophic media in the treated veins, as illustrated in the higher magnification views in Figure 4c,d. There was some alteration of the collagen structure in treated arteries, but no loss of integrity. Perivascular oedema and congested vasa vasorum were also noted. In animal 1, sacrificed 1 day after treatment, luminal arterial defects (focal extensive necrotico-neutrophilic arteritis) and splenic artery obstruction were observed within the irradiated vessel segment, thought to have their traumatic origin due to the extensive endovascular catheter manipulation and balloon over-distension. No compromising effect was observed on the extracellular matrix (ECM) that makes up the integrity of the vessel wall.

## 4. Study Limitations

Several limitations should be acknowledged. First, the vessels treated in this study are not the most clinically relevant in terms of tumour involvement. Most treatments were performed via the splenic vein (*n* = 5), with limited cases involving the splenic artery (*n* = 1) and superior mesenteric artery (SMA, *n* = 1). The splenic vein is the least critical in LAPC resections. This approach was chosen to minimise risk in this initial proof-of-concept phase, as the splenic vein is technically readily accessible and in contact with the pancreas, allowing for quantitative assessment of the extent of necrosis in relation to the administered drug dose.

Second, the follow-up period was limited to 48 h in most animals, restricting assessment of long-term vascular healing and downstream effects on adjacent organs. As such, data on long-term safety is required. Future studies in the porcine model are planned to target more clinically relevant vessels (e.g., celiac trunk, splenic artery, SMA) with extended follow-up of up to two months. These studies will assess the risks of thrombosis, stenosis, perforation, or aneurysm formation and evaluate whether acute vessel wall changes, such as necrotizing arteritis, resolve without clinical consequence, as suggested by Mansfield et al. and others [33,34,35,36,37,38,39,40,41,42,43,44,45,46,47,48,49,50].

Third, this study is primarily a drug dose escalation. Light dose escalation is constrained in the endovascular setting due to limitations in irradiation time (when using occlusive balloons), device power output, and the need to avoid thermal damage. While light fractionation (with intermittent balloon deflation) could extend exposure time, this was initially deemed impractical by clinicians due to extended intraprocedural time; however, it is now under re-evaluation for future studies.

Finally, the use of the normal porcine model limits generalizability. EPA dose responses may differ in human tissue and particularly in pancreatic tumours, due to variations in drug uptake and tissue sensitivity. Currently, large animal models that replicate the vascular anatomy and tumour biology of human pancreatic cancer are not readily available, necessitating cautious progression toward clinical studies.

## 5. Discussion

This study demonstrates that circumferential perivascular necrotic margins can be generated in normal pancreatic tissue using EPA, without causing vessel perforation, bleeding, or compromising vascular integrity up to 48 hrs post-treatment. These findings suggest that clinically relevant EPA light doses can be delivered safely via major vessels.

As with any endovascular procedure, precise technique is critical to avoid complications such as perforation, dissection, or occlusion. This includes minimising device manipulation and ensuring appropriate balloon catheter sizing to prevent over-distension.

Although follow-up was limited to 48 h, prior studies consistently report a low long-term risk of serious vascular complications following EPA [33,34,35,36,37,38,39,40,41,42,43,44,45,46,47,48,49,50]. Nevertheless, photoactivation of non-targeted agents like Verteporfin can affect vascular tissues. Studies designed to assess the limits of vessel wall injury have shown that complete ablation of endothelial and smooth muscle cells is possible [46,47,48]. However, the extracellular matrix (ECM)—primarily collagen and elastin—remains intact, preserving vessel structure. Animal models show that the remaining decellularized scaffold repopulates over time: endothelial cells within weeks, and medial/adventitial layers over months, sometimes incompletely. Despite this, vessels remain patent, with no reports of rupture, thrombosis, stenosis, or aneurysm [46,47,48].

Supporting this, studies using a phthalocyanine—a photo-activated drug with similarities to Verteporfin—on rat carotid arteries demonstrated medial muscle cell ablation without compromising vessel integrity [44]. Follow-up from 3 days to 6 months confirmed structural preservation. In rabbit carotids, mechanical strength testing at 3, 7, and 21 days post-photoactivation showed no reduction in bursting pressure compared to controls. Histology revealed full-thickness cell loss without inflammation, and Elastic van Gieson staining confirmed preservation of elastic laminae and collagen. The authors concluded that photoactivated arteries, despite cellular ablation, are not at risk of perforation, thrombosis, or haemorrhage. Furthermore, several Phase I/II clinical studies investigating vascular photoactivation report consistent safety with follow-up periods up to 51 months [35,36].

In the present study, vascular effects were observed, including endothelial detachment, medial atrophy, and necrotizing arteritis. Whether these changes resolve without clinical consequence in oncological settings—particularly in the context of perivascular necrosis—remains an open question and will be assessed in detail in future studies.

From experimental [26,54] and clinical studies, we know that the selectivity of non-targeted photo-activated drugs between normal and neoplastic tissues is relative, not absolute, and that in many cases, lesions in normal tissue can heal by regeneration of essentially normal tissue. This was demonstrated in the pancreas in the first reported clinical study (which used Foscan (mTHPC) for image-guided, percutaneous, interstitial PDT for pancreatic cancer [29]. In one case, the irradiating fibre slipped after insertion, prior to light delivery, and a zone of necrosis was produced in the normal pancreas. By one month, this had healed without any adverse events (pancreatitis, leak of pancreatic fluids or haemorrhage), providing reassurance that normal pancreas can tolerate such effects. In most cases, areas of treated cancer also healed safely, but in 2 cases, there was post-treatment bleeding, thought to be from the gastrointestinal artery [29], although this could be controlled by non-surgical means. Rather than just abutment, this was most likely due to direct invasion of the blood vessels by the cancer, compromising the extracellular matrix of the blood vessel wall. This is an aspect that must be kept in mind. If there is direct cancer invasion through the vessel wall, EPA is unlikely to be suitable, unless the vessel wall is protected, for example, via stent placement.

If successfully translated into clinical practice, the necrotic margins observed here may enable the creation of a zone free of viable tumour between the vessel wall and viable tumour tissue. This could make surgery feasible for patients previously deemed inoperable due to vascular involvement. The current porcine model provides in vivo proof-of-concept data, supporting further translational research to address these open questions and study limitations.

## 6. Future Outlook

The preliminary results so far are encouraging and warrant additional laboratory investigations to substantiate the current findings and support potential progression to pilot clinical studies.

Such a pilot study may be performed in patients scheduled for left pancreatectomy and splenectomy, where the affected vessels lie entirely within the area to be resected. This allows for an “ablate-and-resect” design, with histological evaluation of the treated vessels and surrounding tissue a few days post-EPA. Such a study could provide critical short-term safety data and histopathological data on the tissue response, without altering standard surgical care.

Given that Verteporfin is already approved and endovascular balloon catheters are widely used in clinical practice, such short-term safety trials could be initiated relatively soon after consolidatory laboratory studies have been completed, for which our group has already started planning. However, longer-term safety data will be essential before broader clinical application.

Long-term survival in pancreatic cancer is often limited by poor chemotherapy efficacy, largely due to the dense desmoplastic stroma [55]. Photo-activated ablation has shown promise in disrupting this barrier, enhancing drug penetration and efficacy for agents such as paclitaxel [56], doxorubicin [57], and irinotecan [58]. While the mechanisms remain under investigation, it is hypothesised that photo-activated ablation increases vascular permeability through oxidative damage to endothelial and stromal cells, and possibly through collagen denaturation [59]. However, care must be taken, as collagen preservation is critical for maintaining vascular integrity.

Moreover, photo-activated ablation may be enhanced through molecular targeting and has been shown to induce damage-associated molecular patterns (DAMPs), potentially priming the immune system and synergizing with immunotherapies [60,61].

## 7. Conclusions

This study demonstrates that EPA can safely induce perivascular necrosis in pancreatic tissue adjacent to major vessels, without compromising vascular integrity when appropriate devices and techniques are used. This approach may enable the creation of a zone free of viable tumour in patients with locally advanced pancreatic cancer involving major vessels, potentially converting inoperable cases into candidates for surgical resection.

EPA holds promise not only for local tumour control but also for overcoming desmoplasia, enhancing chemotherapy delivery, and synergizing with immunotherapy. These findings support further translational research and the design of early-phase clinical trials.

## Figures and Tables

**Table 1:** Observed dose-dependent perivascular necrotic depth in the porcine pancreas, histopathological damage patterns to the vessel, survival period, and EPA dose parameters.

Nr.	Target Vessel Diameter [mm]	Depth of Necrosis Range [mm]	Endothelial Response	Vessel Wall Response	Survival [days]	Drug Dose [mg·kg^−1^]	Light Dose [J·cm^−1^]
1	SMA 7	6–10	Activated	Necrotising arteritis	1	0.4	495
2	Splenic artery 4	0–5	Ulcerated	Necrotising arteritis	2	0.4	540
3	Splenic vein 8	0	No change observed	No change observed	7	0.4	377
4	Splenic vein 8	6–10	Detached	Thickened intima, anthropic media	2	0.8	270
5	Splenic vein 8	6–10	Detached	Thickened intima, anthropic media	2	1.6	311
6	Splenic vein 10	11–15	Detached	Thickened intima, anthropic media	2	3.0	276
7	Splenic vein 10	11–15	Detached	Atrophic media	2	3.2	321

## Data Availability

The data supporting this study’s findings are available from the corresponding author upon reasonable request.

## References

[1] Sung H., Ferlay J., Siegel R.L., Laversanne M., Soerjomataram I., Jemal A., Bray F. (2021). Global Cancer Statistics 2020: GLOBOCAN Estimates of Incidence and Mortality Worldwide for 36 Cancers in 185 Countries. CA A Cancer J. Clin..

[2] Rombouts S.J.E., Vogel J.A., van Santvoort H.C., van Lienden K.P., van Hillegersberg R., Busch O.R.C., Besselink M.G.H., Molenaar I.Q. (2015). Molenaar. Systematic review of innovative ablative therapies for the treatment of locally advanced pancreatic cancer. Br. J. Surg..

[3] Katz M.H., Pisters P.W., Evans D.B., Sun C.C., Lee J.E., Fleming J.B., Vauthey N.J., Abdalla E.K., Crane C.H., Wolff R.A. (2008). Borderline Resectable Pancreatic Cancer: The Importance of This Emerging Stage of Disease. J. Am. Coll. Surg..

[4] Gemenetzis G., Groot V.P., Blair A.B., Laheru D.A., Zheng L., Narang A.K., Fishman E.K., Hruban R.H., Yu J., Burkhart R.A. (2019). Survival in Locally Advanced Pancreatic Cancer After Neoadjuvant Therapy and Surgical Resection. Ann. Surg..

[5] Narayanan G., Daye D., Wilson N.M., Noman R., Mahendra A.M., Doshi M.H. (2021). Ablation in Pancreatic Cancer: Past, Present and Future. Cancers.

[6] Ruarus A., Vroomen L., Puijk R., Scheffer H., Meijerink M. (2018). Locally Advanced Pancreatic Cancer: A Review of Local Ablative Therapies. Cancers.

[7] Schmedt C.-G., Sroka R., Steckmeier S., Meissner O., Babaryka G., Hunger K., Ruppert V., Sadeghi-Azandaryani M., Steckmeier B. (2006). Investigation on Radiofrequency and Laser (980 nm) Effects after Endoluminal Treatment of Saphenous Vein Insufficiency in an Ex-vivo Model. Eur. J. Vasc. Endovasc. Surg..

[8] Heger M., van Golen R.F., Broekgaarden M., Bos R.R.v.D., Neumann H.A.M., van Gulik T.M., van Gemert M.J.C. (2013). van Gemert. Endovascular laser–tissue interactions and biological responses in relation to endovenous laser therapy. Lasers Med. Sci..

[9] Overchuk M., Weersink R.A., Wilson B.C., Zheng G. (2023). Photodynamic and Photothermal Therapies: Synergy Opportunities for Nanomedicine. ACS Nano.

[10] Meyer M. (2019). Processing of collagen based biomaterials and the resulting materials properties. Biomed. Eng. Online.

[11] Ruarus A.H., Vroomen L.G.P.H., Geboers B., van Veldhuisen E., Puijk R.S., Nieuwenhuizen S., Besselink M.G., Zonderhuis B.M., Kazemier G., de Gruijl T.D. (2020). Percutaneous Irreversible Electroporation in Locally Advanced and Recurrent Pancreatic Cancer (PANFIRE-2): A Multicenter, Prospective, Single-Arm, Phase II Study. Radiology.

[12] Bretschneider T., Ricke J., Gebauer B., Streitparth F. (2016). Image-Guided High-Dose-Rate Brachytherapy of Malignancies in Various Inner Organs—Technique, Indications, and Perspectives. J. Contemp. Brachyther..

[13] Omari J., Heinze C., Wilck A., Hass P., Seidensticker M., Seidensticker R., Mohnike K., Ricke J., Pech M., Powerski M. (2019). Efficacy and safety of CT-guided high-dose-rate interstitial brachytherapy in primary and secondary malignancies of the pancreas. Eur. J. Radiol..

[14] Maor E., Ivorra A., Mitchell J.J., Rubinsky B. (2010). Vascular Smooth Muscle Cells Ablation with Endovascular Nonthermal Irreversible Electroporation. J. Vasc. Interv. Radiol..

[15] Montoya M.M., Bustamante T.G., Kulstad E., Mickelsen S., Suarez A.G. (2023). Analysis of thermal effects from pulsed field ablation. Eur. Heart J..

[16] Chun K.-R.J., Miklavčič D., Vlachos K., Bordignon S., Scherr D., Jais P., Schmidt B. (2024). State-of-the-art pulsed field ablation for cardiac arrhythmias: Ongoing evolution and future perspective. EP Eur..

[17] Desai V., Sampieri G., Namavarian A., Lee J.M. (2023). Cryoablation for the treatment of chronic rhinitis: A systematic review. J. Otolaryngol.—Head Neck Surg..

[18] Sun X., Zhao S., Yu S., Cui K. (2023). Cryoballoon vs. Laser Balloon Ablation for Atrial Fibrillation: A Meta-Analysis. Front. Cardiovasc. Med..

[19] Tzeis S., Gerstenfeld E.P., Kalman J., Saad E.B., Shamloo A.S., Andrade J.G., Barbhaiya C.R., Baykaner T., Boveda S., Calkins H. (2024). 2024 European Heart Rhythm Association/Heart Rhythm Society/Asia Pacific Heart Rhythm Society/Latin American Heart Rhythm Society expert consensus statement on catheter and surgical ablation of atrial fibrillation. EP Eur..

[20] Taylor R.J., Matthews G.J., Aseltine R.H., Fields E.C. (2024). Clinical outcomes in borderline and locally advanced pancreatic cancer with the addition of low-dose-rate brachytherapy to standard of care therapy. Brachytherapy.

[21] Willink C.Y., Jenniskens S.F.M., Klaassen N.J.M., Stommel M.W.J., Nijsen J.F.W. (2023). Intratumoural injection therapies for locally advanced pancreatic cancer: Systematic review. BJS Open.

[22] Jermyn M., Davis S.C., Dehghani H., Huggett M.T., Hasan T., Pereira S.P., Bown S.G., Pogue B.W. (2014). CT contrast predicts pancreatic cancer treatment response to verteporfin-based photodynamic therapy. Phys. Med. Biol..

[23] Huggett M.T., Jermyn M., Gillams A., Illing R., Mosse S., Novelli M., Kent E., Bown S.G., Hasan T., Pogue B.W. (2014). Phase I/II study of verteporfin photodynamic therapy in locally advanced pancreatic cancer. Br. J. Cancer.

[24] Hanada Y., Pereira S.P., Pogue B., Maytin E.V., Hasan T., Linn B., Mangels-Dick T., Wang K.K. (2021). EUS-guided verteporfin photodynamic therapy for pancreatic cancer. Gastrointest. Endosc..

[25] Moesta K.T., Schlag P., Douglass H.O., Mang T.S. (1995). Evaluating the role of photodynamic therapy in the management of pancreatic cancer. Lasers Surg. Med..

[26] Ayaru L., Wittmann J., MacRobert A., Novelli M., Bown S.G., Pereira S.P. (2007). Photodynamic Therapy Using Verteporfin Photosensitization in the Pancreas and Surrounding Tissues in the Syrian Golden Hamster. Pancreatology.

[27] Karimnia V., Slack F.J., Celli J.P. (2021). Photodynamic Therapy for Pancreatic Ductal Adenocarcinoma. Cancers.

[28] Abulafi A.M., Allardice J.T., Williams N.S., van Someren N., Swain C.P., Ainley C. (1995). Photodynamic therapy for malignant tumours of the ampulla of Vater. Gut.

[29] Bown S.G., Rogowska A.Z., Whitelaw D.E., Lees W.R., Lovat L.B., Ripley P., Jones L., Wyld P., Gillams A., Hatfield A.W.R. (2002). Photodynamic therapy for cancer of the pancreas. Gut.

[30] Choi J.-H., Oh D., Lee J.H., Park J.-H., Kim K.-P., Lee S.S., Lee Y.-J., Lim Y.-S., Song T.J., Seo D.-W. (2015). Initial human experience of endoscopic ultrasound-guided photodynamic therapy with a novel photosensitizer and a flexible laser-light catheter. Endoscopy.

[31] DeWitt J.M., Sandrasegaran K., O’Neil B., House M.G., Zyromski N.J., Sehdev A., Perkins S.M., Flynn J., McCranor L., Shahda S. (2019). Phase 1 study of EUS-guided photodynamic therapy for locally advanced pancreatic cancer. Gastrointest. Endosc..

[32] Huggett M.T., Jermyn M., Gillams A., Mosse S., Kent E., Bown S.G., Hasan T., Pogue B.W., Pereira S.P. (2013). Photodynamic therapy of locally advanced pancreatic cancer (VERTPAC study): Final clinical results. Optical Methods for Tumor Treatment and Detection: Mechanisms and Techniques in Photodynamic Therapy XXII.

[33] Jenkins M.P., Buonaccorsi G.A., Raphael M., Nyamekye I., McEwan J.R., Bown S.G., Bishop C.C.R. (1999). Clinical study of adjuvant photodynamic therapy to reduce restenosis following femoral angioplasty. Br. J. Surg..

[34] Kereiakes D.J., Szyniszewski A.M., Wahr D., Herrmann H.C., Simon D.I., Rogers C., Kramer P., Shear W., Yeung A.C., Shunk K.A. (2003). Phase I Drug and Light Dose-Escalation Trial of Motexafin Lutetium and Far Red Light Activation (Phototherapy) in Subjects With Coronary Artery Disease Undergoing Percutaneous Coronary Intervention and Stent Deployment. Circulation.

[35] Mansfield R.J.R., Jenkins M.P., Pai M.L., Bishop C.C.R., Bown S.G., McEwan J.R. (2002). Long-term safety and efficacy of superficial femoral artery angioplasty with adjuvant photodynamic therapy to prevent restenosis. Br. J. Surg..

[36] Rockson S.G., Kramer P., Razavi M., Szuba A., Filardo S., Fitzgerald P., Cooke J.P., Yousuf S., DeVault A.R., Renschler M.F. (2000). Photoangioplasty for Human Peripheral Atherosclerosis. Circulation.

[37] Usui M., Miyagi M., Fukasawa S., Hara T., Ueyama N., Nakajima H., Takata R., Sasame A., Tamura K., Naitou Y. (2004). A first trial in the clinical application of photodynamic therapy for the prevention of restenosis after coronary-stent placement. Lasers Surg. Med..

[38] Grant W., Speight P., MacRobert A., Hopper C., Bown S. (1994). Photodynamic therapy of normal rat arteries after photosensitisation using disulphonated aluminium phthalocyanine and 5-aminolaevulinic acid. Br. J. Cancer.

[39] Grant W.E., Buonaccorsi G., Speight P.M., Macrobert A.J., Hopper C., Bown S.G. (1995). The effect of photodynamic therapy on the mechanical integrity of normal rabbit carotid arteries. Laryngoscope.

[40] Nyamekye I., Anglin S., McEwan J., MacRobert A., Bown S., Bishop C. (1995). Photodynamic Therapy of Normal and Balloon-Injured Rat Carotid Arteries Using 5-Amino-Levulinic Acid. Circulation.

[41] Jenkins M.P., Buonaccorsi G., MacRobert A., Bishop C.C.R., Bown S.G., McEwan J.R. (1998). Intra-arterial photodynamic therapy using 5-ALA in a swine model. Eur. J. Vasc. Endovasc. Surg..

[42] Jenkins M. (2000). Reduction in the response to coronary and iliac artery injury with photodynamic therapy using 5-aminolaevulinic acid. Cardiovasc. Res..

[43] Nagae T., Aizawa K., Uchimura N., Tani D., Abe M., Fujishima K., Wilson S.E., Ishimaru S. (2001). Endovascular photodynamic therapy using mono-L-aspartyl-chlorin e6 to inhibit Intimal hyperplasia in balloon-injured rabbit arteries. Lasers Surg. Med..

[44] Nyamekye I., Buonaccorsi G., McEwan J., MacRobert A., Bown S., Bishop C. (1996). Inhibition of intimal hyperplasia in balloon injured arteries with adjunctive phthalocyanine sensitised photodynamic therapy. Eur. J. Vasc. Endovasc. Surg..

[45] Ortu P., LaMuraglia G.M., Roberts W.G., Flotte T.J., Hasan T. (1992). Photodynamic therapy of arteries. A novel approach for treatment of experimental intimal hyperplasia. Circulation.

[46] LaMuraglia G.M., ChandraSekar N.R., Flotte T.J., Abbott W.M., Michaud N., Hasan T. (1994). Photodynamic therapy inhibition of experimental intimal hyperplasia: Acute and chronic effects. J. Vasc. Surg..

[47] Waksman R., McEwan P.E., Moore T.I., Pakala R., Kolodgie F.D., Hellinga D.G., Seabron R.C., Rychnovsky S.J., Vasek J., Scott R.W. (2008). PhotoPoint Photodynamic Therapy Promotes Stabilization of Atherosclerotic Plaques and Inhibits Plaque Progression. J. Am. Coll. Cardiol..

[48] Jain M., Zellweger M., Frobert A., Valentin J., van Den Bergh H., Wagnières G., Cook S., Giraud M.-N. (2016). Intra-Arterial Drug and Light Delivery for Photodynamic Therapy Using Visudyne^®^: Implication for Atherosclerotic Plaque Treatment. Front. Physiol..

[49] Hsiang Y.N., Crespo M.T., Richter A.M., Jain A.K., Fragoso M., Levy J.G. (1993). In vitro and in vivo uptake of benzoporphyrin derivative into human and miniswine atherosclerotic plaque. Photochem. Photobiol..

[50] Allison B.A., Crespo M.T., Jain A.K., Richter A.M., Hsiang Y.N., Levy J.G. (1997). Delivery of Benzoporphyrin Derivative, a Photosensitizer, into Atherosclerotic Plaque of Watanabe Heritable Hyperlipidemic Rabbits and Balloon-Injured New Zealand Rabbits. Photochem. Photobiol..

[51] Saeidi T., García Vázquez A., Verde J.M., Wanert F., Spiridon I.A., Schmid A., Swanstrom L., Bown S.G., Lilge L., Bogaards A. (2025). Dose Threshold Values for Endovascular Photodynamic Therapy (PDT) in Normal Pig Pancreas and Human Pancreatic Cancer. Photochem. Photobiol. Scie..

[52] Nichols A.B., Smith R., Berke A.D., Shlofmitz R.A., Powers E.R. (1989). Importance of balloon size in coronary angioplasty. J. Am. Coll. Cardiol..

[53] Kobayashi N., Hirano K., Yamawaki M., Araki M., Sakai T., Sakamoto Y., Mori S., Tsutsumi M., Honda Y., Ito Y. (2018). Simple classification and clinical outcomes of angiographic dissection after balloon angioplasty for femoropopliteal disease. J. Vasc. Surg..

[54] Mlkvy P., Messman H., MacRobert A., Pauer M., Sams V.R., Davies C.L., Stewart J.C.M., Bown S. (1997). Photodynamic therapy of a transplanted pancreatic cancer model using meta-tetrahydroxyphenyl chlorin (MTHPC). Br. J. Cancer.

[55] Adiseshaiah P.P., Crist R.M., Hook S.S., McNeil S.E. (2016). Nanomedicine strategies to overcome the pathophysiological barriers of pancreatic cancer. Nat. Rev. Clin. Oncol..

[56] Araki T., Ogawara K.-I., Suzuki H., Kawai R., Watanabe T.-I., Ono T., Higaki K. (2015). Augmented EPR effect by photo-triggered tumor vascular treatment improved therapeutic efficacy of liposomal paclitaxel in mice bearing tumors with low permeable vasculature. J. Control. Release.

[57] Luo D., Carter K.A., Molins E.A.G., Straubinger N.L., Geng J., Shao S., Jusko W.J., Straubinger R.M., Lovell J.F. (2019). Pharmacokinetics and pharmacodynamics of liposomal chemophototherapy with short drug-light intervals. J. Control. Release.

[58] Huang H.-C., Rizvi I., Liu J., Anbil S., Kalra A., Lee H., Baglo Y., Paz N., Hayden D., Pereira S. (2018). Photodynamic Priming Mitigates Chemotherapeutic Selection Pressures and Improves Drug Delivery. Cancer Res..

[59] Gutierrez N.M.C., Pujol-Solé N., Arifi Q., Coll J.-L., le Clainche T., Broekgaarden M. (2022). Increasing cancer permeability by photodynamic priming: From microenvironment to mechanotransduction signaling. Cancer Metastasis Rev..

[60] Bhandari C., Moffat A., Shah N., Khan A., Quaye M., Fakhry J., Soma S., Nguyen A., Eroy M., Malkoochi A. (2024). PD-L1 Immune Checkpoint Targeted Photoactivable Liposomes (iTPALs) Prime the Stroma of Pancreatic Tumors and Promote Self-Delivery. Adv. Healthc. Mater..

[61] Saad M.A., Zhung W., Stanley M.E., Formica S., Grimaldo-Garcia S., Obaid G., Hasan T. (2022). Photoimmunotherapy Retains Its Anti-Tumor Efficacy with Increasing Stromal Content in Heterotypic Pancreatic Cancer Spheroids. Mol. Pharm..

